# Histone deacetylase 6 acts upstream of DNA damage response activation to support the survival of glioblastoma cells

**DOI:** 10.1038/s41419-021-04182-w

**Published:** 2021-09-28

**Authors:** Wen-Bin Yang, An-Chih Wu, Tsung-I Hsu, Jing-Ping Liou, Wei-Lun Lo, Kwang-Yu Chang, Pin-Yuan Chen, Ushio Kikkawa, Shung-Tai Yang, Tzu-Jen Kao, Ruei-Ming Chen, Wen-Chang Chang, Chiung-Yuan Ko, Jian-Ying Chuang

**Affiliations:** 1 TMU Research Center of Neuroscience, Taipei Medical University, 11031 Taipei, Taiwan; 2 Graduate Institute of Medical Sciences, College of Medicine, Taipei Medical University, 11031 Taipei, Taiwan; 3 The Ph.D. Program for Neural Regenerative Medicine, College of Medical Science and Technology, Taipei Medical University, 11031 Taipei, Taiwan; 4 TMU Research Center of Cancer Translational Medicine, Taipei Medical University, 11031 Taipei, Taiwan; 5 Cell Physiology and Molecular Image Research Center, Wan Fang Hospital, Taipei Medical University, 11031 Taipei, Taiwan; 6 School of Pharmacy, College of Pharmacy, Taipei Medical University, 11031 Taipei, Taiwan; 7 TMU Research Center of Drug Discovery, Taipei Medical University, 11031 Taipei, Taiwan; 8 Department of Neurosurgery, Shuang Ho Hospital, Taipei Medical University, 23561 New Taipei City, Taiwan; 9 Department of Surgery, School of Medicine, College of Medicine, Taipei Medical University, 11031 Taipei, Taiwan; 10 National Institute of Cancer Research, National Health Research Institutes, 70456 Tainan, Taiwan; 11 Department of Neurosurgery, Keelung Chang Gung Memorial Hospital, 20401 Keelung, Taiwan; 12 Department of Biomedical Science and Environmental Biology, Kaohsiung Medical University, 80708 Kaohsiung, Taiwan

**Keywords:** Cancer therapeutic resistance, Mechanisms of disease, CNS cancer

## Abstract

DNA repair promotes the progression and recurrence of glioblastoma (GBM). However, there remain no effective therapies for targeting the DNA damage response and repair (DDR) pathway in the clinical setting. Thus, we aimed to conduct a comprehensive analysis of DDR genes in GBM specimens to understand the molecular mechanisms underlying treatment resistance. Herein, transcriptomic analysis of 177 well-defined DDR genes was performed with normal and GBM specimens (*n* = 137) from The Cancer Genome Atlas and further integrated with the expression profiling of histone deacetylase 6 (HDAC6) inhibition in temozolomide (TMZ)-resistant GBM cells and patient-derived tumor cells. The effects of HDAC6 inhibition on DDR signaling were examined both in vitro and intracranial mouse models. We found that the expression of DDR genes, involved in repair pathways for DNA double-strand breaks, was upregulated in highly malignant primary and recurrent brain tumors, and their expression was related to abnormal clinical features. However, a potent HDAC6 inhibitor, MPT0B291, attenuated the expression of these genes, including RAD51 and CHEK1, and was more effective in blocking homologous recombination repair in GBM cells. Interestingly, it resulted in lower cytotoxicity in primary glial cells than other HDAC6 inhibitors. MPT0B291 reduced the growth of both TMZ-sensitive and TMZ-resistant tumor cells and prolonged survival in mouse models of GBM. We verified that HDAC6 regulated DDR genes by affecting Sp1 expression, which abolished MPT0B291-induced DNA damage. Our findings uncover a regulatory network among HDAC6, Sp1, and DDR genes for drug resistance and survival of GBM cells. Furthermore, MPT0B291 may serve as a potential lead compound for GBM therapy.

## Introduction

Glioblastoma (GBM), the most common adult malignant brain tumor, is one of the deadliest cancers, as it involves a highly aggressive feature and causes poor prognosis even after simultaneous standard treatment with radiation and temozolomide (TMZ)-based chemotherapy [1, 2]. TMZ is an imidazotetrazine derivative of an alkylating agent and acts as a DNA methylating agent, thereby resulting in DNA lesion (O6-methylguanine, N7-methylguanine, and N3-methyladenine) and DNA mismatches to cause cellular senescence and apoptosis [3]. Promoter methylation of the DNA repair gene, O-6-methylguanine-DNA methyltransferase (MGMT), which acts by removing alkyl groups from DNA, has been associated with longer survival in response to TMZ treatment [4]. Induction of MGMT expression is frequently associated with TMZ treatment at recurrence. However, resistance to TMZ also occurs in MGMT-negative GBM cells, indicating the involvement of other factors [3, 5].

In clinical practice, patients with isocitrate dehydrogenase (NADP(+)) 1 (IDH1)^R132H^ mutation, a diagnostic marker and prognostic indicator of GBM, gain survival benefit in contrast to those without the mutation in both primary and secondary GBM [6, 7]. IDH1 is a key enzyme that catalyzes the conversion of isocitrate to α-ketoglutaric acid (αKG) in the tricarboxylic acid cycle. IDH1^R132H^ mutation acquires neomorphic ability to produce D-2-hydroxyglutarate from αKG, causing cellular alterations resulting in metabolic dysfunction and inhibition of DNA repair [6]. Several studies shed light on the impact of IDH1^R132H^ mutation with impaired DNA repair mechanisms, including inhibition of DNA repair enzyme alkB homolog 1, histone H2A dioxygenase (ALKBH) as well as defects of homologous recombination (HR) DNA repair [8, 9]. HR is one of the two major DNA double-strand break (DSB) repair pathways, which repairs broken DNA caused by oncogene-induced replication stress, ionizing radiation, or chemotherapeutic drugs [10]. DNA damage response and repair (DDR) signaling is considered to be a barrier for tumor formation and drug resistance [11, 12]. These findings provide a clue that targeting the general DDR signaling is crucial as IDH1-mutated features may be potential targets in most patients with GBM.

Several abnormally expressed-DDR genes, including exonuclease 1 (EXO1), nei-like DNA glycosylase 3 (NEIL3), and DNA damage-binding protein 2 (DDB2), are associated with GBM progression and susceptibility [13]. Poly-ADP-ribose polymerase (PARP) is an abundant nuclear enzyme involved in base excision repair (BER), and a PARP inhibitor is currently administered in adult patients with unresectable or partially resectable GBM in a clinical trial [14]. The clinical effectiveness of therapies targeting these genes is, however, still rare. Namely, there is a lack of a systemic approach that integrates the genome-wide landscape of expression profiling, big data analytics, and clinical significance, which might be critical issues in translational research for the application of DNA repair blockade.

Histone deacetylase 6 (HDAC6) belongs to the class IIb of HDAC family and is regulated by several protein kinases, including protein kinase C alpha [15], extracellular signal-regulated kinase [16], G protein-coupled receptor kinase 2 [17], casein kinase 2 [18], aurora A kinase [19], etc., which lead to HDAC6 activation and its downstream targets in altering multiple cellular processes. HDAC6 is considered to play a unique role owing to its cytoplasmic localization and ability to deacetylate non-histone proteins [20]. HDAC6 affects the dynamics of the cellular structure through deacetylating α-tubulin in the assembled microtubules [21]. Recent studies have highlighted the importance of the nuclear functions of HDAC6 in GBM progression and recurrence. HDAC6, which is overexpressed in clinical GBM tumors [22], TMZ-resistant GBM cells and GBM stem-like tumorspheres [23], affects the expression of cell-cycle-related genes and cancer stemness-related genes through regulating acetylation levels of Sp1 transcription factor [23, 24]. Furthermore, it has been reported that HDAC6 deacetylates mutS homolog 2 (MSH2), a key DNA mismatch repair (MMR) protein [25]. Inhibition of HDAC6 correlates with increased expression of MSH2 in TMZ-resistant GBM cells [26]; however, whether HDAC6 controls function in DNA repair through MMR remains unknown. Further, there is still a lack of comprehensive analysis to elucidate the mechanism of HDAC6-mediated DNA repair signaling in GBM progression and recurrence. Therefore, in this study, we collected human GBM transcriptome data from The Cancer Genome Atlas (TCGA-GBM dataset) and performed a systemic analysis for characterizing the functional role of HDAC6 in DDR pathways, including not only HR, BER, MMR, but also nucleotide excision repair (NER), nonhomologous end joining (NHEJ), etc. Also, we used in vitro experiments and intracranial mouse models for targeting HDAC6 via pharmacological approaches and genetic knockdown to further examine the role of HDAC6 on the DDR regulation in both TMZ-sensitive and TMZ-resistant GBM.

## Results

### Inhibition of HDAC6 reverses abnormal clinical features of DDR genes in GBM

**Fig. 1:**
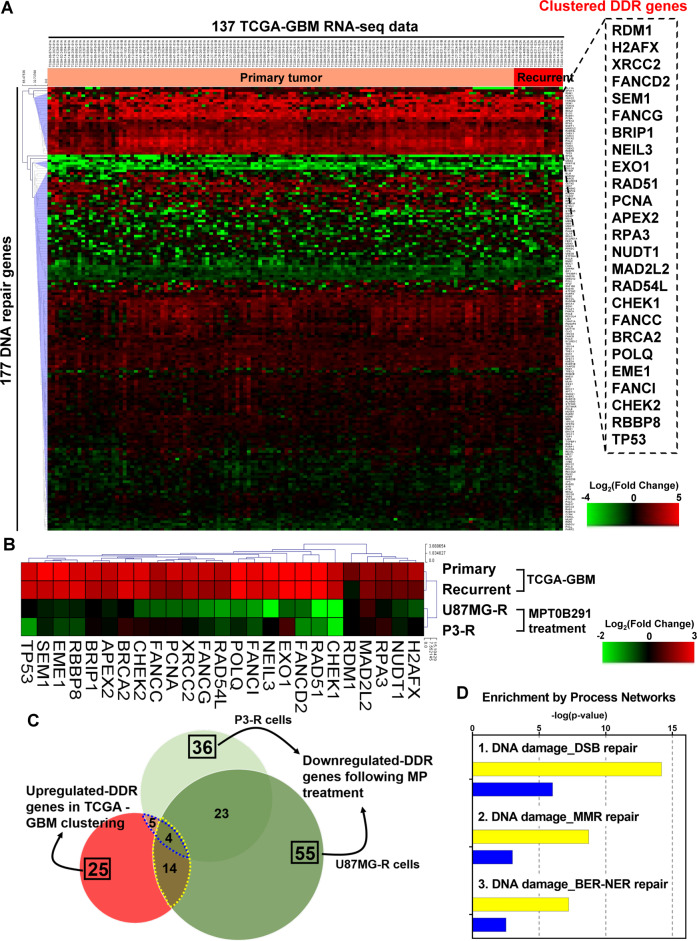
MPT0B291 reverses abnormal clinical features of DDR genes in GBM. **A** Heatmap representation of the expression levels of 177 well-defined DDR genes in 137 clinical primary or recurrent GBM samples from the TCGA database. Twenty-five upregulated genes (the clustered DDR genes) compared to solid normal controls were clustered using the Euclidean distance method and shown in the dashed frame on the right panel. **B** Heatmap representation of fold changes of the clustered DDR genes identified by the above analysis in primary and recurrent tumors versus normal brain tissue and in MPT0B291-treated U87MG-R and P3-R cells versus DMSO-treated parental cells. **C** Venn diagram showing overlaps between the number of DDR genes that were upregulated (two-fold increase) in TCGA-GBM dataset or downregulated (1.5-fold decrease) after MPT0B291 treatment in U87MG-R and P3-R cells. **D** MetaCore analysis was conducted on the intersected genes between TCGA-GBM dataset and MPT0B291-treated U87MG-R/P3-R cells according to the above analysis to evaluate their functions. The yellow and blue bars show results of 14 and 5 co-regulated DDR genes, respectively. The number of involved genes is shown on the bars.

To investigate the DDR gene expression in clinical GBM tumors, we collected RNA-seq data of 137 GBM samples from the TCGA database. By further analysis of 177 well-defined DDR genes using hierarchical clustering, we identified 25 DDR genes (the clustered DDR genes) that were significantly (*p* < 0.05) upregulated in primary and recurrent GBM (Fig. 1A).

Our recent study have indicated that MPT0B291, a potent HDAC6 inhibitor, induces G2/M arrest and senescence in both parental and TMZ-resistant GBM cells [23]. Here, we further analyzed whether the pathway of “DNA replication, recombination, and repair” is underlying MPT0B291-induced growth inhibition of GBM cells. The DDR gene expression profiling following the MPT0B291 treatment of TMZ-resistant U87MG (U87MG-R) or patient-derived P3 (P3-R) cells was performed. The comparison of the expression of DDR genes between MPT0B291-treated cells and TCGA-GBM dataset revealed a negative correlation (Supplementary Fig. S1A, PCC = −0.272), especially in the clustered DDR genes (Supplementary Fig. S1B, PCC = −0.412). The reduction of the DDR gene expression was observed following MPT0B291 treatment (Fig. 1B), in which 14 and 5 DDR genes in U87MG-R and P3-R cells, respectively (Fig. 1C) exhibited converse changes of those of the clustered DDR genes shown in Fig. 1A. Enrichment analysis of the overlapping genes using MetaCore software (Fig. 1D) identified that the top three process networks were “DSB repair”, “MMR repair”, and “BER-NER repair”. These results suggest that HDAC6 may be involved in regulating the abnormal expression of the DDR genes affecting DNA repair.

### Inhibition of HDAC6 decreases DDR gene expression, induces DNA damage, and inhibits the growth of GBM and TMZ-resistant GBM

**Fig. 2:**
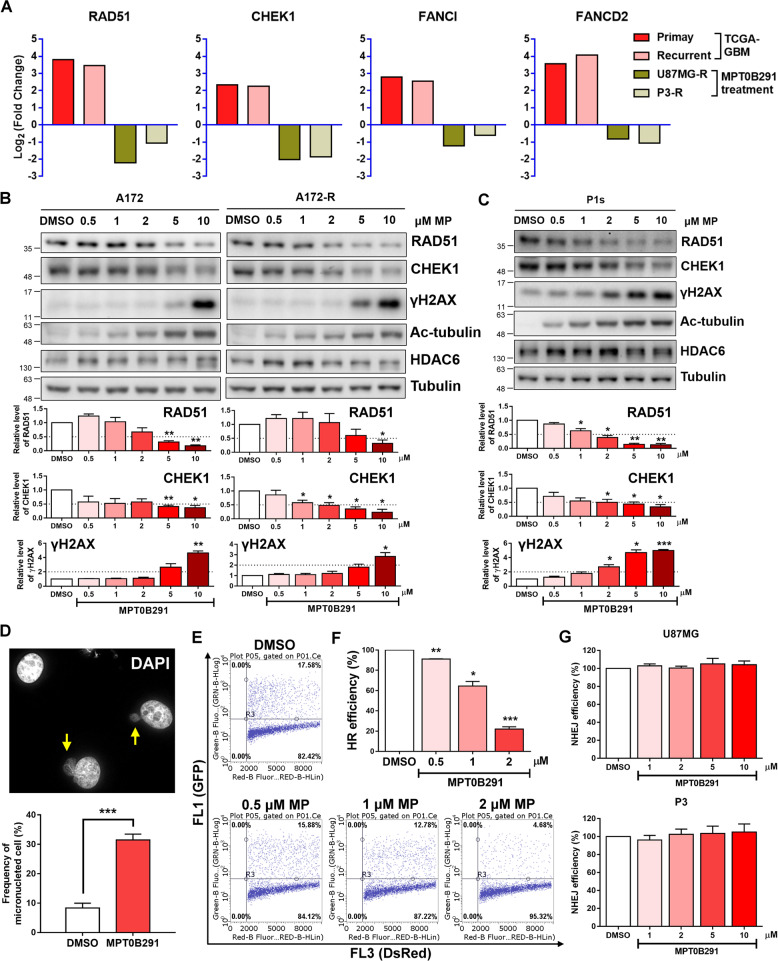
MPT0B291 decreases DDR gene expression and induces DNA damage. **A** The fold change in the expression of RAD51, CHEK1, FANCI, and FANCD2 in TCGA-GBM dataset of primary and recurrent tumor samples, and in MPT0B291-treated U87MG-R and P3-R cells. **B** A172, A172-R, and **C** P1s cells were treated with the indicated doses of MPT0B291 for 24 h, and the protein levels of RAD51, CHEK1, γH2AX, Ac-tubulin, HDAC6, and tubulin were analyzed by western blotting (upper panels). Quantitative results (normalized to tubulin) of RAD51, CHEK1, and γH2AX from three independent experiments are shown (lower panels). **D** U87MG cells were fixed and stained with DAPI following DMSO or MPT0B291 treatment for 24 h. The number of micronuclei (indicated by arrows) was calculated and quantified in more than 600 cells from three independent experiments. **E** HR assay was performed after the treatment with DMSO or indicated doses of MPT0B291. The percentage of **F** HR and **G** NHEJ efficiency was calculated as the ratio of MPT0B291 treatment to DMSO treatment and quantified after three independent experiments.

**Fig. 3:**
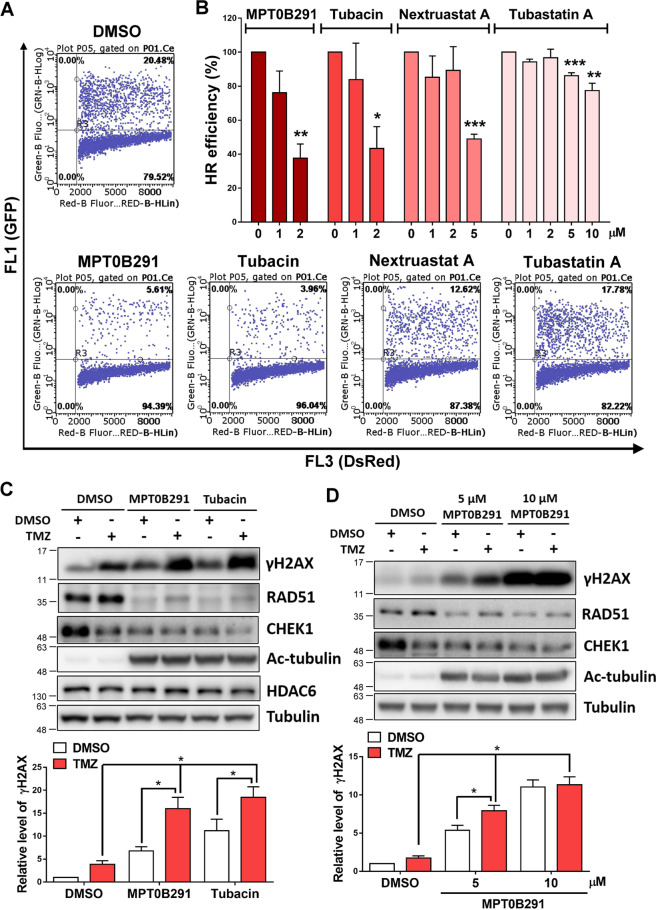
Inhibition of HDAC6 enhances TMZ-induced DNA damage response. **A, B** HR assay was performed after the treatment with DMSO or each HDAC6 inhibitor for 48 h at 2 μM in **A** and at indicated doses in **B**. Percentage of HR efficiency was calculated as the ratio of the indicated dosage of HDAC6 inhibitor treatment to DMSO treatment and quantified after three independent experiments. **C** U87MG cells were treated with 5 μM MPT0B291 or tubacin in the presence (100 μM) or absence of TMZ for 24 h. **D** A172 cells were treated with the indicated concentrations of MPT0B291 in the presence (100 μM) or absence of TMZ for 24 h. The protein levels of γH2AX, RAD51, CHEK1, HDAC6, Ac-tubulin, and tubulin were analyzed by western blotting (upper panels). Quantitative results (normalized to tubulin) of γH2AX from three independent experiments are shown (lower panels).

Among the significantly altered genes overlapped in TCGA-GBM dataset and MPT0B291-treated GBM cells, four genes (RAD51, CHK1, FANCI, and FANCD2) were the most potential ones involved in the regulation of HDAC6-mediated DNA repair (Fig. 2A). Furthermore, the protein expression of RAD51 recombinase (catalyzes the core reactions of HR) and CHEK1 (a kinase central for the DDR signaling at the S and G2/M cell cycle checkpoints) was examined, and dose-related decreases of both protein levels were observed after MPT0B291 treatment in A172 GBM cells and TMZ-resistant A172 GBM cells (Fig. 2B), as well as in the patient-derived TMZ-resistant GBM cells (Fig. 2C). Also, we found a corresponding increase in the levels of phospho-histone H2A.X (γH2AX, a marker for DNA damage) and acetyl-α-tubulin (ac-tubulin, a marker for HDAC6 inhibition) in these cells (Fig. 2B, C). An increase in the frequency of micronuclei cells relative to the control group (Fig. 2D) indicated that MPT0B291 induces unrepaired DSB or mitotic spindle damage. Supporting this finding, we observed that MPT0B291 impaired DSB repair by blocking HR repair (Fig. 2E, F) rather than by NHEJ (Fig. 2G). To avoid the off-target effects of MPT0B291, we also confirmed the HR efficiency after other commercial HDAC6 inhibitor treatment (including tubacin, nextruastat A, and tubastatin A). All of the HDAC6 inhibitors could significantly decrease the repair efficiency of HR. However, MPT0B291 and tubacin showed more effective inhibition of HR at 2 μM concentration. (Fig. 3A, B). DDR genes (RAD51, CHEK1, and γH2AX) had the same expression patterns in MPT0B291- and tubacin-treated cells (Fig. 3C). Surprisingly, combined treatment with a lower dosage of HDAC6 inhibitor and TMZ was more effective in triggering DDR than individual treatment (Fig. 3C, D). However, treatment with a higher dosage (10 μM) of MPT0B291 could fully induce DDR whether co-treated with TMZ or not (Fig. 3D), suggesting that MPT0B291 is highly effective in inducing DNA damage and is an ideal choice for a single-drug treatment. These findings provide a regulatory mechanism of DNA repair by HDAC6 because DNA damaging agents are widely used in oncology to treat many cancers; therefore, targeting HDAC6 could be a therapeutic strategy for GBM.

**Fig. 4:**
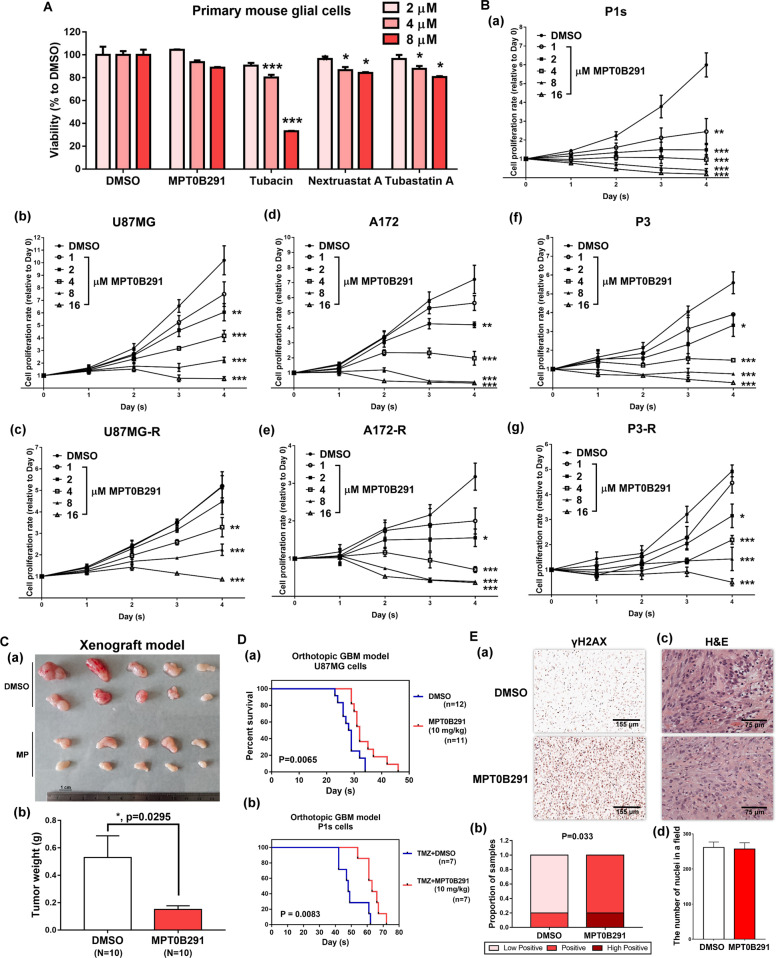
MPT0B291 inhibits the growth of GBM and TMZ-resistant GBM. **A** Primary mouse glial cells were treated with DMSO or indicated concentrations of MPT0B291, tubacin, nexturastat A, and tubastatin A for 2 days. After treatment, the proportion of surviving cells was estimated using the MTT assay. Quantitative result (relative to DMSO) from three independent experiments is shown. **B** P1s cells (a), U87MG (b), U87MG-R (c), A172 (d), A172-R (e), P3 (f), and P3-R (g) were treated with the indicated concentrations of MPT0B291 for 4 days. After treatment, the proportion of surviving cells was estimated using the MTT assay. Data (relative to Day 0) are representative of three independent experiments and presented as the mean ± SEM. **C** Tumors with DMSO or 10 mg/kg MPT0B291 treatment from SCID mice implanted with U87MG cells for 6 weeks are shown (a), and quantitative result of tumor weights is shown (b). **D** U87MG (a) or P1s cells (b) inoculated orthotopic mice were randomly grouped and treated with DMSO, 10 mg/kg TMZ (for P1s cells inoculated orthotopic mice) or 10 mg/kg MPT0B291 three days/week thereafter from day 5. Results of the Kaplan–Meier curve for the duration of survival in the control group (blue line) and MPT0B291-treated group (red line) are shown. **E** γH2AX levels in xenograft tumors were studied using IHC staining (a), and quantified (b) using ImageJ analysis with the IHC profiler score (*n* = 10). H&E staining of xenograft tumors (c) for nuclei counting using ImageJ analysis (d).

We further confirmed the effectiveness of MPT0B291 in GBM treatment. MPT0B291 at concentrations of 2–8 μM did not induce significant cell toxicity in primary glial cells (Fig. 4A); however, it inhibited cell proliferation and induced cell death in both GBM, TMZ-resistant GBM, and patient-derived TMZ-resistant GBM cells (Fig. 4B). In addition, attenuation of tumor growth was noted in the MPT0B291-treated group (Fig. 4C). MPT0B291 also prolonged mouse survival in orthotopic transplantation models of GBM cells, including TMZ-sensitive and TMZ-resistant cells (Fig. 4D). Activation of the DDR signal could be detected in the MPT0B291-treated group (Fig. 4E). These results indicate that HDAC6 is an attractive therapeutic target in brain tumors.

### The HDAC6/Sp1 axis mediates the abnormal regulation of DDR genes in GBM

**Fig. 5:**
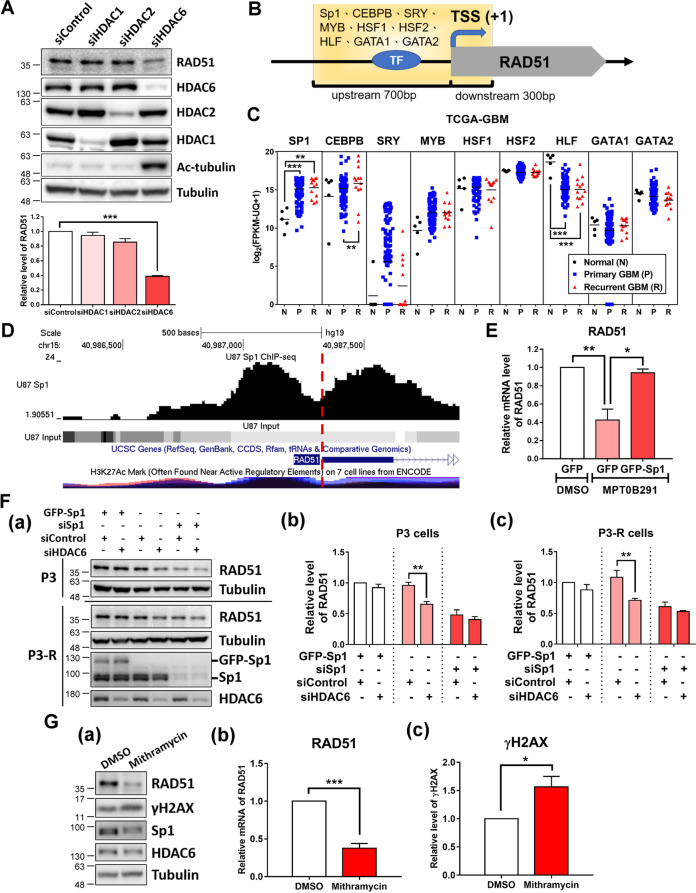
HDAC6 regulates RAD51 expression through Sp1. **A** U87MG cells were transfected with control, HDAC1, HDAC2, and HDAC6 siRNA, respectively, for 72 h. The protein levels of RAD51, HDAC1, HDAC2, HDAC6, Ac-tubulin, and tubulin were analyzed by western blotting (upper panels). Result of the quantitative analysis (normalized to tubulin) of RAD51 from three independent experiments is shown (lower panel). **B** Schematic diagram shows the promoter region used for transcription factor-binding analysis. TSS transcription start site. **C** The mRNA expression levels of SP1, CEBPB, SRY, MYB, HSF1, HSF2, HLF, GATA1, and GATA2 in normal brain, primary, and recurrent tumor samples (TCGA-GBM dataset). **D** Distributions of Sp1-ChIP-seq reads mapped to the promoter region of RAD51. Visualization of the Sp1-ChIP-Seq data using the UCSC Genome Browser on Human (hg19) Assembly (https://genome.ucsc.edu/). The red dash line indicates TSS. **E** U87MG cells were treated with DMSO or 10 μM MPT0B291 following transfection with GFP or GFP-Sp1 plasmid. On the next day of treatment, the mRNA expression of RAD51 in cells was analyzed using qPCR. Quantitative result (normalized to GAPDH) from three independent experiments is shown. **F** P3 and P3-R cells were transfected with GFP-Sp1, siSp1, siControl, and siHDAC6, respectively, for 72 h. The protein levels of RAD51, Sp1, HDAC6, and tubulin were analyzed using western blotting (a). Quantitative results (normalized to tubulin) of RAD51 in P3 cells (b) and P3-R cells (c) from three independent experiments are shown. **G** P3-R cells were treated with 10 μM mithramycin A for 24 h. The protein levels of RAD51, γH2AX, Sp1, HDAC6, and tubulin were analyzed by western blotting (a). Quantitative results (normalized to tubulin) of RAD51 (b) and γH2AX (c) from three independent experiments are shown.

Although MPT0B291 is a potent and selective HDAC6 inhibitor, with an IC_50_ value of 5 nM, it can also target HDAC1 and HDAC2 at higher treatment concentrations [23]. To elucidate which HDAC is involved in regulating the expression of DDR genes, we used the knockdown approach with siRNA to directly target HDAC1, HDAC2, or HDAC6. Knockdown of HDAC6 significantly decreased the expression of RAD51 but that of HDAC1 and HDAC2 did not (Fig. 5A). This result led to further investigation of possible transcription factors (TFs) involved in the regulation of HDAC6-mediated DDR genes. For this purpose, we integrated promoter analysis of dysregulated DDR genes, and expression profiles of TFs in the TCGA-GBM dataset to identify potential TFs. The transcription factor Sp1 was the most potential TF involved in regulating the abnormal expression of DDR genes, which commonly existed in the promoter of DDR genes and was significantly upregulated in primary and recurrent GBMs (Supplementary Fig. S2). Using the same analysis approach, we observed that Sp1 was also one of the candidate TFs involved in the regulation of RAD51 (Fig. 5B, C).

Our recent study has indicated that MPT0B291-mediated Sp1 acetylation shows a decreased DNA binding ability of Sp1 resulting in altered expression of downstream genes [23]. In addition, we have found that the depletion of Sp1 significantly decreases HR efficiency [27]; however, the detailed mechanism is unknown. Analysis of Sp1-ChIP-seq data revealed that Sp1 is bound on the promoter region of RAD51 (Fig. 5D). Overexpression of Sp1 reversed the effect of MPT0B291-mediated RAD51 downregulation (Fig. 5E). Furthermore, knockdown of HDAC6 downregulated RAD51 expression in both P3 and P3-R cells; however, it did not decrease significantly in Sp1-overexpressed and -knockdown cells (Fig. 5F). The expression of RAD51 was also downregulated in mithramycin A (Sp1 inhibitor)-treated cells and an accompanied increase was observed in γH2AX levels (Fig. 5G). These results indicate that Sp1 is involved in the regulation of DDR by controlling the expression level of RAD51.

**Fig. 6:**
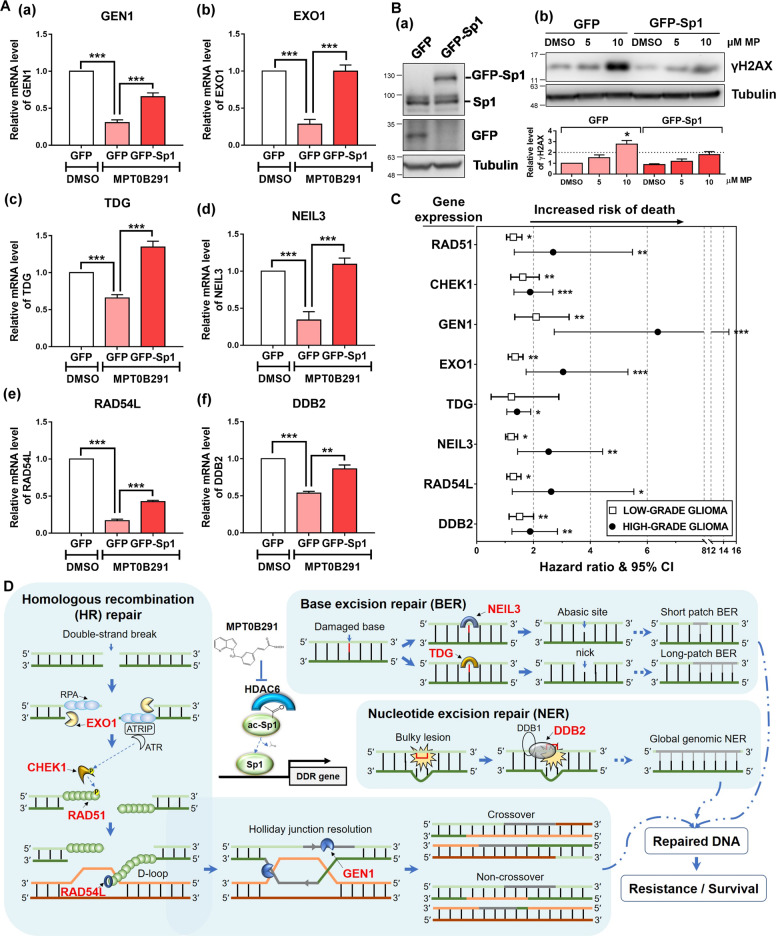
Sp1 regulates DDR gene expression and impairs MPT0B291-induced DNA damage. **A** U87MG cells were treated with DMSO or 10 μM MPT0B291 following transfection with GFP or GFP-Sp1 plasmid. One day after treatment, the mRNA expression of GEN1 (a), EXO1 (b), TDG (c), NEIL3 (d), RAD54L (e), and DDB2 (f) in cells was analyzed by qPCR. Quantitative results (normalized to GAPDH) from five independent experiments are shown. **B** A172 cells were transfected with GFP or GFP-Sp1, and treated with the indicated dosage of MPT0B291 for 24 h. The protein levels of GFP-Sp1, Sp1, GFP (a), γH2AX and tubulin (b) were analyzed by western blotting. Quantitative result (normalized to tubulin) of γH2AX from three independent experiments is shown (lower panel). **C** Forest plots showing hazard ratios for the risk of death in patients with low-grade and high-grade glioma having higher expression of the indicated gene(s). The lines on both sides denote 95% confidence intervals. All the original data (Kaplan–Meier curve) were obtained from PROGgeneV2 database [51] (Supplementary Fig. S6). Hazard ratios above one indicate a poor outcome. **D** Schematic diagram shows that MPT0B291 induces DNA damage and cell death through the HDAC6/Sp1-mediated DDR gene regulation.

The HDAC6/Sp1 axis may not target the RAD51 promoter alone. In addition to RAD51, 13 DDR gene promoter sequences were also occupied by Sp1 (Supplementary Fig. S3). We integrated multiomics data from the Sp1-binding profile, and gene expression profiles of clinical GBM samples (TCGA-GBM dataset) and MPT0B291-treated GBM cells. We identified that 8 DDR genes (including RAD51, CHEK1, GEN1, EXO1, TDG, NEIL3, RAD54L, and DDB2) shows a potential to be regulated by HDAC/Sp1 (Supplementary Table S1). Supporting this finding, the expression of these DDR genes was significantly downregulated by MPT0B291 in both GBM and TMZ-resistant GBM cells (Fig. 6A and Supplementary Fig. S4). Moreover, the effects of MPT0B291-mediated DDR gene inhibition (Fig. 6A) and DNA damage induction (Fig. 6B) were impaired in Sp1-overexpressed cells. The HDAC6 signaling leading to DDR gene expression is also confirmed by the knockout approach with HDAC6 to overcome the limitation of the chemical inhibition of HDAC6 using MPT0B291 (Supplementary Fig. S5). Further analysis of the clinical outcome data showed that higher expression levels of these DDR genes in high-grade and/or low-grade gliomas are significantly associated with decreased survival (Fig. 6C and Supplementary Fig. S6). Taken together, these results highlight the importance of the HDAC6/Sp1 axis in the DDR pathway, suggesting that targeting HDAC6/Sp1 signaling is an effective strategy against GBM and recurrent GBM with higher DNA repair capacity.

## Discussion

Induction of DNA damage has served as a therapeutic strategy for several cancers. Apart from chemotherapeutic drugs that exert cytotoxic effects, developing targeted therapy against DDR signaling is one of the current treatment strategies. There are two types of Food and Drug Administration-approved DDR inhibitors (PARP inhibitor and topoisomerase inhibitors), which are applied in cancer therapy [28]. Olaparib (Lynparza), a PARP inhibitor that has recently been approved for the treatment of metastatic breast cancer harboring BRCA1 or BRCA2 mutations, represents the first medicine based on the precision medicine concept. The median progression-free survival of patients with HER2-negative and BRCA mutation receiving Lynparza is 7 months compared to that of 4.2 months in patients taking chemotherapy alone [29]. Therefore, understanding the patients’ genetic makeup or gene expression profile is crucial in clinical practice.

Recent advances in genomics, bioinformatics, and big biodata analysis have been the driving forces to implement precision medicine in clinical practice [30]. Biological databases such as Gene Expression Omnibus and TCGA integrate and provide enormous amounts of omics data, serving as crucially important resources for scientists [31]. To elucidate the gene expression profiles in patients with GBM, we obtained transcriptome NGS data from TCGA. The TCGA-GBM dataset was obtained from a total of 142 specimens: 5 normal tissues, 124 primary tumor tissues, and 13 recurrent tumor tissues. After performing systematic analysis on the 177 well-defined DDR genes, we identified several DDR genes that were dysregulated in clinical GBM cases. Even though some of the DDR genes, such as enhancer of zeste 2 polycomb repressive complex 2 subunit (EZH2) and human telomerase reverse transcriptase (hTERT), have been recently reported to be altered in primary GBM [11], most studies focus on one of the DDR genes of their interest. Since cancer is a complex disease, changes in multiple gene expression contribute to tumor progression and the concept of multitarget drugs has also been applied in the clinical setting [32]. Understanding pathway-targeted-drug–disease relationships is required. To this end, we performed bioinformatics studies to identify the promoter region of GBM-associated DDR genes for detection of upstream regulators. We had found that Sp1 is most potential to regulate DDR gene expression, and this result explains the mechanism how Sp1 regulates DNA repair in GBM [27]. A detailed study of HDAC6/Sp1-DDR gene regulation may provide new insights into GBM therapy because targeting this pathway may have an equal effect to that of multitarget drugs. Indeed, Sp1 depletion by Sp1 or HDAC6 inhibitors resulted in decreased expression of RAD51, CHEK1, EXO1, RAD54L, and GEN1, and then caused defective HR repair. Additionally, signaling of BER and NER is potentially influenced through the downregulation of NEIL3, TDG, and DDB2 expression (Fig. 6D). These gene expression statuses were also strongly correlated with the prognosis of the glioma patients (Fig. 6C); particularly, low expression levels were observed in patients with IDH1 mutation (Supplementary Fig. S7). Although IDH1 mutation has dual effects, acting either as an oncogene or as a tumor suppressor gene in glioma [7, 33], patients with IDH mutation exhibited better prognosis than those with wild-type IDH1 [34, 35] (Supplementary Fig. S8). Inhibition of the HDAC6/Sp1 signaling pathway could reverse DDR gene expression from the wild-type form to the mutated form of IDH1 and exhibited better prognosis in the clinical setting. Taken together, the HDAC6/Sp1 axis is a critical pathway toward advancing treatment for GBM.

Disruption of HDAC activities has been achieved with targeted therapy in different cancer types [36]. In GBM clinical trials, including SAHA combined with chemoradiotherapy for newly diagnosed GBM (NCT00731731), as well as SAHA plus bevacizumab (NCT01738646), panobinostat (PS) plus bevacizumab (NCT00859222), and romidepsin (NCT00085540) for recurrent GBM, had not improved the progression-free survival at 6 months or median overall survival. However, several HDAC inhibitor-associated clinical trials are still ongoing. In addition to the development of the isoform- or class-specific HDAC inhibitors, advances in brain-targeted delivery strategies could be considered important for the treatment of patients with GBM. Many challenges exist, such as cancer stemness, the blood–brain barrier (BBB) and drug-efflux pumps. More recently, we have provided evidence that a novel HDAC6 inhibitor, MPT0B291, inhibits the growth of stem-like/drug-resistant GBM cells and has better BBB permeability values than caffeine [23]. Thus, MPT0B291 may be a good treatment option for patients with GBM. A recent study showed that reversal of gene expression abnormalities correlates with drug efficacy in several diseases including breast, liver, and colon cancers [37]. Although Gobin et al. have identified DNA repair and cell cycle gene expression signatures for the classification of clinical samples using the nCounter technology [38], they have not provide any candidate compound that could be applied for clinical use. In the present study, we provided a list of abnormal DDR gene expression in GBM using the TCGA-GBM dataset big biodata analytics. Comparison of the expression of these genes between MPT0B291-treated samples and patients with GBM revealed a negative correlation (Pearson’s r = −0.412) (Supplementary Fig. S1B), indicating that inhibition of HDAC6 by MPT0B291 has a potential to reverse the abnormalities of DDR genes in GBM. In more detail, RAD51, showed to be the most influenced DDR gene following MPT0B291 treatment (Fig. 2A), has been associated with radioresistance in GBM stem cells [39]. HDAC inhibitors, including SAHA and valproic acid (VPA), could reduce the expression levels of RAD51 [40]; however, the detailed mechanisms remain unclear. In the present study, we found that only HDAC6 inhibition can downregulate RAD51 expression through Sp1-mediated transcriptional regulation (Fig. 5). A study on acute myelogenous leukemia has indicated that HDAC inhibition by SAHA or PS results in the downregulation of RAD51 through miR-182 regulation [41]. It is also reported that Sp1 can also regulate miR-182 expression [42]. These observations raise the possibility that RAD51 seems to be controlled by Sp1 at both transcriptional and post-transcriptional levels, highlighting the importance of Sp1 in RAD51-mediated HR repair.

In conclusion, our study uncovers the HDAC6/Sp1 signaling axis as an important pathway for the protection of GBM cells against DNA damage. HDAC6 inhibitors may serve as potential lead compounds for GBM therapy. These data could provide a foundation for clinical practice in the future.

## Materials and methods

### Cell culture

All cells, including U87MG (ATCC HTB-14, Manassas, VA), A172 (ATCC CRL-1620), P3 (patient-derived primary GBM) [23], and their respective TMZ-resistant cells, as well as P1s (patient-derived chemo-resistant GBM) and primary mouse glial cells, were maintained in Dulbecco’s modified Eagle’s medium (DMEM; Gibco, Waltham, MA) supplemented with 10% fetal bovine serum (Gibco) at 37 °C in a humidified atmosphere containing 5% CO_2_. Details of the isolation of patient-derived GBM cells are described previously [23, 27].

HDAC6 knockout (KO)-U87MG cells (commercialized KO cell line was prepared using clustered regularly interspaced short palindromic repeats strategy under the assistance of Biotools Co., Ltd. (New Taipei City, Taiwan)).

For preparing primary mouse glial cell cultures, cortices from postnatal day 0 (P0) to P1 mouse pups were dissociated by trituration and digestion with 10 U/ml trypsin (Gibco) for 30 min at 37 °C, and filtered through a 70 µm nylon mesh cell strainer (Corning Incorporated, Corning, NY). Cells were plated onto cell culture plates coated with 50 μg/ml poly-L-lysine (Sigma–Aldrich, Temecula, CA). The mixed glial cultures were purified one week after isolation. Culture plates were taped onto a shaker inside an incubator and shaken at 150 rpm for 2 h. The supernatant was removed and cells were maintained in the DMEM culture medium. This procedure allowed cultures with a mixture of primary glial cells. Experiments are conducted under a protocol approved by the Joint Institutional Review Board of Taipei Medical University (Taipei, Taiwan) with the registration numbers (Nos. 201006011 and 201402018) and by the Institutional Animal Care and Use Committee (IACUC) of National Health Research Institutes (NHRI; Miaoli, Taiwan) with the registration number (NHRI-IACUC-106010).

### Dataset collection and processing

The publicly available next-generation sequencing (NGS) databases of human GBM transcriptome were obtained from TCGA (https://portal.gdc.cancer.gov). The FPKM-UQ values of 177 well-defined DDR genes (these genes were obtained from the DDRprot database [43]) expressed in all of the 142 specimens, which consisted of five normal, 124 primary tumor, and 13 recurrent tumor tissues, were selected for the analysis. The fold change in gene expression and the level of significance of DDR genes between normal and GBM tissues were calculated. Details of ChIP-seq profiling of Sp1 in U87MG and expression profiling of 177 well-defined DDR genes in MPT0B291-treated U87MG-R or P3-R cells were acquired from our previous study [23].

### Bioinformatics analysis

For cluster analysis of DDR gene expression profiling and upstream transcription factor-binding profiling, hierarchical clustering was performed using the Euclidean distance method and average linkage [44]. Pathway and process network analyses were performed using MetaCore software (Clarivate Analytics, Philadelphia, PA). For upstream transcription factor-binding analysis, the promoter sequences (1000 bp upstream to 200 bp downstream) of the clustered DDR genes (defined in Results) were obtained from the GenBank database and analyzed using TFBIND tools [45]. The criterion of matrix similarity was >0.9.

### Western blotting

Sample lysates were prepared and western blotted as described previously [23]. Antibodies used were as follows: RAD51 [D4B10] rabbit monoclonal antibody (mAb) (#8875, Cell Signaling Technology, Danvers, MA); CHEK1 [C1C2-6] rabbit polyclonal antibody (polyAb) (GTX100070, GeneTex, Hsinchu, Taiwan); γH2AX [EP854(2)Y] rabbit mAb (ab81299, Abcam, Cambridge, UK); Ac-tubulin [D20G3] rabbit mAb (#5335, Cell Signaling Technology); HDAC6 [D2E5] rabbit mAb (#7558, Cell Signaling Technology); tubulin [1E4C11] mouse mAb (#66031-1-Ig, Proteintech, Rosemont, IL); HDAC1 rabbit polyAb (H3284, Sigma–Aldrich); HDAC2 [3F3] mouse mAb (#05-814, Millipore, Temecula, CA); Sp1 rabbit polyAb (#07-645, Millipore); GFP rabbit polyAb (#632592, Clontech, Mountain View, CA) and horseradish peroxidase-conjugated secondary antibodies (Santa Cruz Biotechnology, Heidelberg, Germany). The protein bands were detected using the ChemiDoc Touch Imaging System (Bio-Rad Laboratories, Hercules, CA) and recorded using Image Lab software (Bio-Rad Laboratories). The band intensities were quantified using Scion image software (Scion, Frederick, MD).

### Micronucleus assay

U87MG cells were seeded onto coverslips (with a thickness of 0.17 mm) and incubated with dimethyl sulfoxide (DMSO; Sigma–Aldrich) or MPT0B291 for 24 h. After fixation with 4% paraformaldehyde (Sigma–Aldrich) in PBS for 15 min and permeabilization with 1% Triton X-100 (Sigma–Aldrich) for 5 min, cells were mounted in ProLong Gold Antifade Mountant with DAPI (Invitrogen, Waltham, MA), and then photographed using an immunofluorescence microscope (Leica DM6000 B; Wetzlar, Germany).

### HR and NHEJ DNA repair assays

For the HR assay [46], cells stably expressing HR reporter plasmid (DR-GFP) were used to measure the HR frequency. The DR-GFP cells were co-transfected at a 4:1 ratio using the I-SceI endonuclease (cleavage creates a DSB) and DsRed-Monomer (as control for transfection efficiency) plasmids. For the NHEJ assay [47], the NHEJ reporter was generated from the GFP vector by NheI (New England Biolabs, Ipswich, Massachusetts) enzyme digestion. U87MG cells were co-transfected at a 2:1 ratio using the NHEJ reporter and DsRed-Monomer plasmids. Cells were harvested 2 days after transfection and subjected to flow cytometry analysis by Guava EasyCyte System (Millipore). Only DsRed-positive cells were analyzed for HR and NHEJ efficiency to circumvent possible differences in transfection efficiencies. Data were analyzed to reveal the percentage of GFP-positive cells relative to that of DsRed-positive cells. Data were set to 1% of the background level of GFP-positive cells in every internal control.

### MTT cell viability assay

Cells were seeded onto a 24-well petri-dish at a density of 1.5 × 104 cells/well. One day after culture (Day 0), the cells were treated with MPT0B291 at varying doses as indicated. For 3-(4,5-dimethylthiazol-2-yl)−2,5-diphenyltetrazolium bromide (MTT; Bionovas, Ontario, Canada) staining, the cells were incubated with fresh medium containing the MTT reagent (final concentration 0.5 mg/ml) at 37 °C for 20 min, and then, the MTT medium was exchanged using 300 µl of DMSO to dissolve the MTT formazan crystals in cells. The absorbance of the supernatant of DMSO extract was measured at a wavelength of 550 nm with a reference wavelength of 655 nm using the iMark Microplate Absorbance Reader (Bio-Rad Laboratories).

### Experimental animals

The animal experiments were approved by the IACUC (NHRI-IACUC-106010) at the NHRI. Five- to six-week-old male immunodeficient NOD.CB17-Prkdc^scid^/JNarl (SCID) mice were purchased from BioLASCO (Taipei, Taiwan) and maintained at the animal facility of NHRI. For subcutaneous inoculation, U87MG (10^6^ cells) were suspended in 100 μl of DMEM and implanted into the back of SCID mice. Tumor weight was determined from tumor tissue surgically excised from the back of SCID mice on day 42 following implantation. For the generation of an orthotopic model, a skull burr hole was created in the right frontal brain area. An ultrafine needle was then inserted to a depth of 3 mm using a stereotactic guiding device, and then, 5 × 10^5^ U87MG or P1s cells (suspended in 3 μl of DMEM) were injected slowly to the mouse brain. In each experiment, all animals received surgically implanted GBM cells from the same researchers and on the same day. Total operating time of surgical procedures in 20 mice are controlled generally for 2 h and GBM cells were placed on the ice to avoid time effects on cell viability. Animals in the same cages were randomly and equally assigned to treatments to avoid cage effects and other possible biases linked to the timing of implantation and intervention. Administration of MPT0B291, TMZ, or vehicle (DMSO) via an intraperitoneal injection was initiated on day 5 after implantation (MPT0B291 was administered 8 h after TMZ injection in the P1s implanted group) and three times per week throughout the duration of the experiment. The scheduled treatment was interrupted when body weight loss was >10% and re-initiated after weight recovery.

### Immunohistochemistry

Xenograft tumors were fixed in 10% formaldehyde for 48 h, dehydrated and embedded in paraffin. Sections were cut and stained with hematoxylin and eosin (H&E). Moreover, Iimmunohistochemical (IHC) analysis was performed, the details of which are described in our previous study [48]. Briefly, blocked histological sections were stained using anti-γH2AX antibody. Immunoreactivity was detected using the DAB substrate kit (Vector Laboratories, Burlingame, CA). The expression level was quantified using the IHC profiler plugin in ImageJ software [49].

### Transient transfection

Cells were used for transfection with plasmids (the manufacturing process has been described previously [50]) including pEGFP (GFP) and pEGFP-Sp1 (GFP-Sp1) using the GenJet reagent (SignaGen Laboratories, Rockville, MD) and commercial gene-specific SMARTpool short-interfering RNAs (Dharmacon, Lafayette, CO) for HDAC1 (siHDAC1), HDAC2 (siHDAC2), HDAC6 (siHDAC6), Sp1 (siSp1), and nontargeting siRNAs control (siControl) using Lipofectamine RNAiMAX (Invitrogen) according to the manufacturer’s protocol. The transfection efficiency was confirmed by western blotting analysis.

### Quantitative PCR

Total RNA was isolated using the TRIzol Reagent (Invitrogen) and Direct-zol RNA Miniprep Kits (Zymo Research, Irvine, CA) according to the manufacturer’s protocol, and 1 μg of RNA was reverse transcribed using the PrimeScript™ RT Reagent Kit (TaKaRa, Mountain View, CA). Quantitative PCR (qPCR) was then performed using a mixture containing cDNA with SYBR Green Master Mix (Bio-Rad Laboratories) and gene-specific primers (Genomics, New Taipei City, Taiwan): RAD51, forward primer (F), 5′-GGGGTGGAGGTGAAGGAAAG-3′ and reverse primer (R), 5′-TGTTCTGTAAAGGGCGGTGG-3′; GEN1, F, 5′-AGCCCCACCTCAGGA ACTTA-3′ and R, 5′-GCACACATGGCTTCAGCTTC-3′; EXO1, F, 5′-CGGGCCAACAATACCTTCCT-3′ and R, 5′-TTGAATGGGCAGGCATAGCA-3′; TDG, F, 5′-TGCCAGAAGAAGTTCCAGCC-3′ and R, 5′-ATCGGGGAGAGTCTTGGTCA-3′; NEIL3, F, 5′-GCCTCTGCATTCTCCGAGTT-3′ and R, 5′-CCCATTTTCTGCCCACTGGA-3′; RAD54L, F, 5′-CGAGCATTGGGCCTGAAAAG-3′ and R, 5′-TGAGGCCGCAAAACCTTACT-3′; DDB2, F, 5′-CCCTTTGACAGGAGGGCTAC-3′ and R, 5′-AACTTCAGCCCAGTGATGCT-3′; GAPDH, F, 5′-GAGTCAACGGATTTGGTCGT-3′ and R, 5′-TTGATTTTGGAGGGATCTCG-3′. mRNA expression levels of the indicated genes were measured using the StepOnePlus™ Real-Time PCR System (Applied Biosystems, Waltham, MA). The relative mRNA level was calculated using the comparative cycle threshold (Ct) method (ΔΔCt) and normalized with the expression of GAPDH.

### Statistical analyses

Statistical analyses of two groups of data from western blotting, micronucleus assay, HR/NHEJ DNA repair assays, MTT assay, tumor weight of subcutaneously inoculated mice, RNA-seq data of TCGA-GBM dataset, qPCR, etc. were carried out using Student’s *t*-test with a two-tailed distribution. Multiple groups of data from the MTT assay and western blotting were analyzed by two-way analysis of variance with subsequent Tukey’s multiple comparison test. The Gehan-Breslow-Wilcoxon test was used to compare the survival curves (Kaplan–Meier curve) of orthotopic GBM mice and patients with high-grade or low-grade gliomas. The Chi-square test was used to analyze IHC results. The DDR gene expression relationships between TCGA-GBM-dataset and MPT0B291-treated GBM cells were examined using the Pearson’s correlation coefficient (PCC). An analysis of variance test is performed to determine whether genes (the RNA sequencing data related to glioma in Chinese Glioma Genome Atlas database) with significantly altered expression in the IDH1 mutant with respect to wild-type. Quantitative data (bar chart) are shown as mean ± SEM. A value of *p* < 0.05 was considered statistically significant (*p < 0.05; **p < 0.01; ***p < 0.001).

## Supplementary information

Supplementary Figure S1

Supplementary Figure S2

Supplementary Figure S3

Supplementary Figure S4

Supplementary Figure S5

Supplementary Figure S6

Supplementary Figure S7

Supplementary Figure S8

Supplementary Table S1

## Data Availability

All data supporting the findings of this study are available within the article and supplementary data.
