# Supplementary material for: Histone deacetylase 6 acts upstream of DNA damage response activation to support the survival of glioblastoma cells
Source: Cell Death Dis. 2021 Sep 28;12(10):884. doi: 10.1038/s41419-021-04182-w (PMC8479077; doi:10.1038/s41419-021-04182-w)
Supplement: Supplementary file 1 — Supplementary Figure S1 [file 41419_2021_4182_MOESM1_ESM.docx]

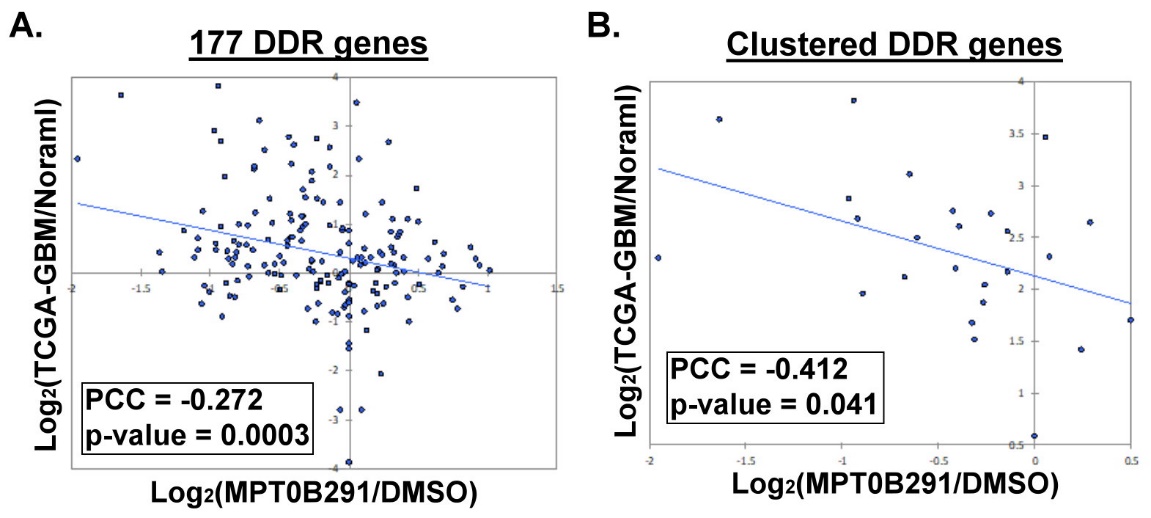


**Supplementary Figure S1. A negative correlation of gene expression profiles between clinical GBM samples and MPT0B291-treated samples.** The scatter plot shows the log_2_ ratio of expression level for (A) 177 DDR genes or (B) 25 DDR genes (the clustered DDR genes) in MPT0B291-treated samples (x-axis) and clinical GBM samples (y-axis). PCC: Pearson's correlation coefficient.
