# Supplementary material for: Histone deacetylase 6 acts upstream of DNA damage response activation to support the survival of glioblastoma cells
Source: Cell Death Dis. 2021 Sep 28;12(10):884. doi: 10.1038/s41419-021-04182-w (PMC8479077; doi:10.1038/s41419-021-04182-w)
Supplement: Supplementary file 2 — Supplementary Figure S2 [file 41419_2021_4182_MOESM2_ESM.docx]

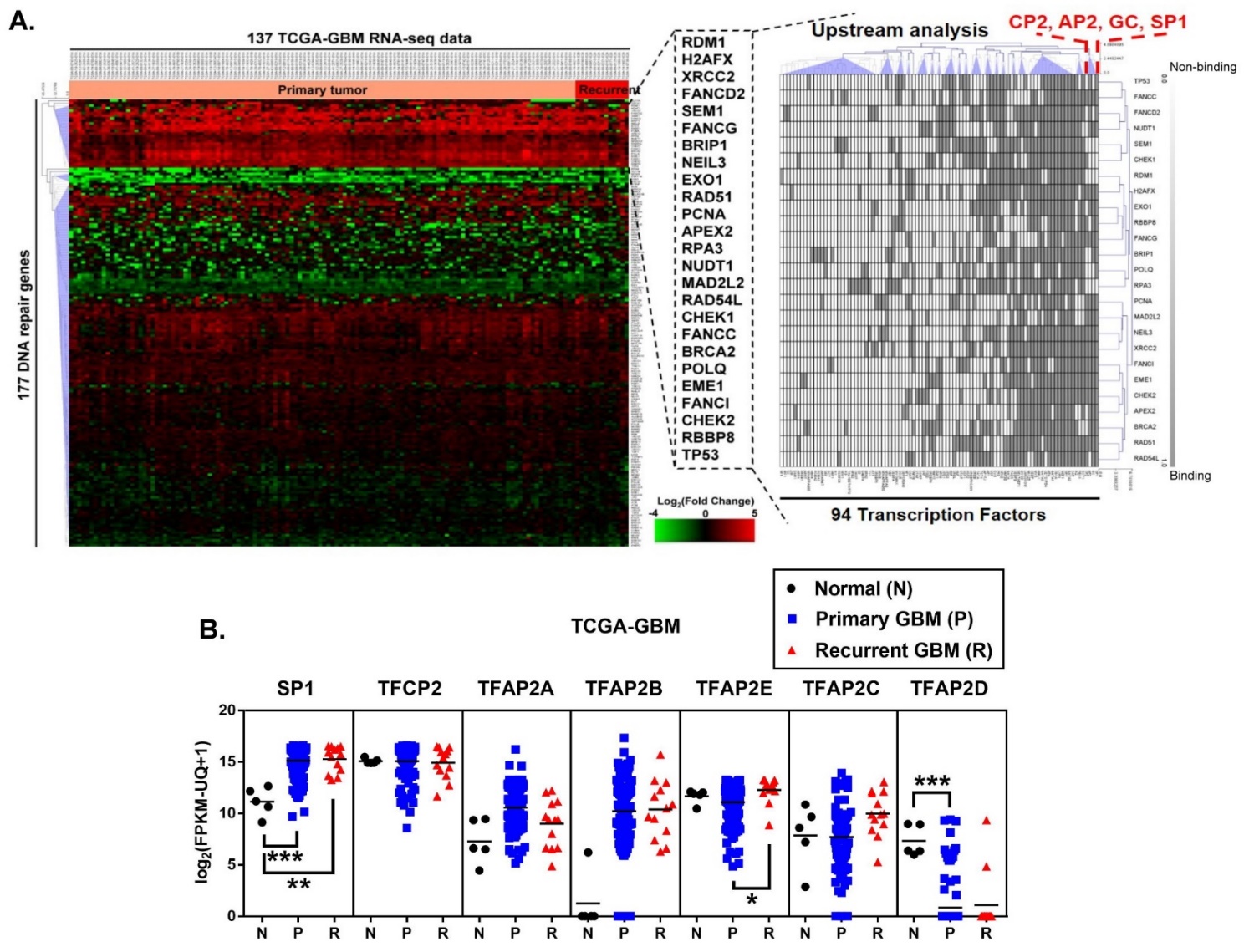


**Supplementary Figure S2.** **Identification of upstream transcription regulators from GBM clinical dataset.** (A) Heatmap representation of the expression level of 177 well-defined DDR genes on 137 of clinical primary or recurrent GBM samples from TCGA database (as shown in Figure 1A). The results of upstream analysis are shown in right panel. (B) The expression levels of Sp1, CP2 (TFCP2), and AP2 (including TFAP2A, TFAP2B, TFAP2E, TFAP2C and TFAP2D) in normal brain, primary, and recurrent tumor samples (TCGA-GBM dataset).
