# Supplementary material for: Histone deacetylase 6 acts upstream of DNA damage response activation to support the survival of glioblastoma cells
Source: Cell Death Dis. 2021 Sep 28;12(10):884. doi: 10.1038/s41419-021-04182-w (PMC8479077; doi:10.1038/s41419-021-04182-w)
Supplement: Supplementary file 3 — Supplementary Figure S3 [file 41419_2021_4182_MOESM3_ESM.docx]

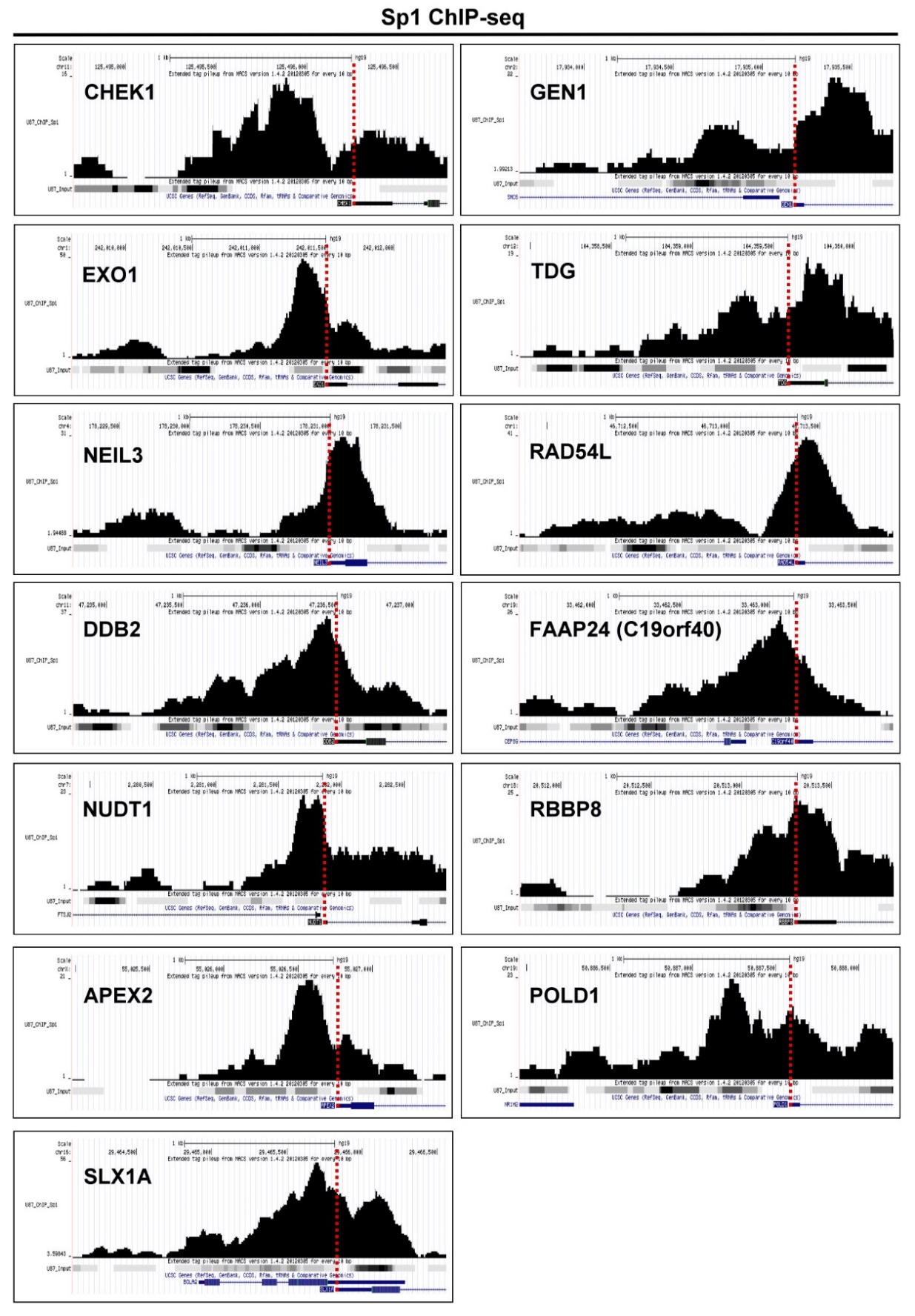


**Supplementary Figure S3. Distributions of Sp1 ChIP-seq reads mapped to promoter region of DDR genes.** Visualizations of Sp1 ChIP-Seq data in U87MG cells using UCSC Genome Browser on Human (hg19) Assembly. Red dash line indicates TSS.
