# Supplementary material for: Histone deacetylase 6 acts upstream of DNA damage response activation to support the survival of glioblastoma cells
Source: Cell Death Dis. 2021 Sep 28;12(10):884. doi: 10.1038/s41419-021-04182-w (PMC8479077; doi:10.1038/s41419-021-04182-w)
Supplement: Supplementary file 4 — Supplementary Figure S4 [file 41419_2021_4182_MOESM4_ESM.docx]

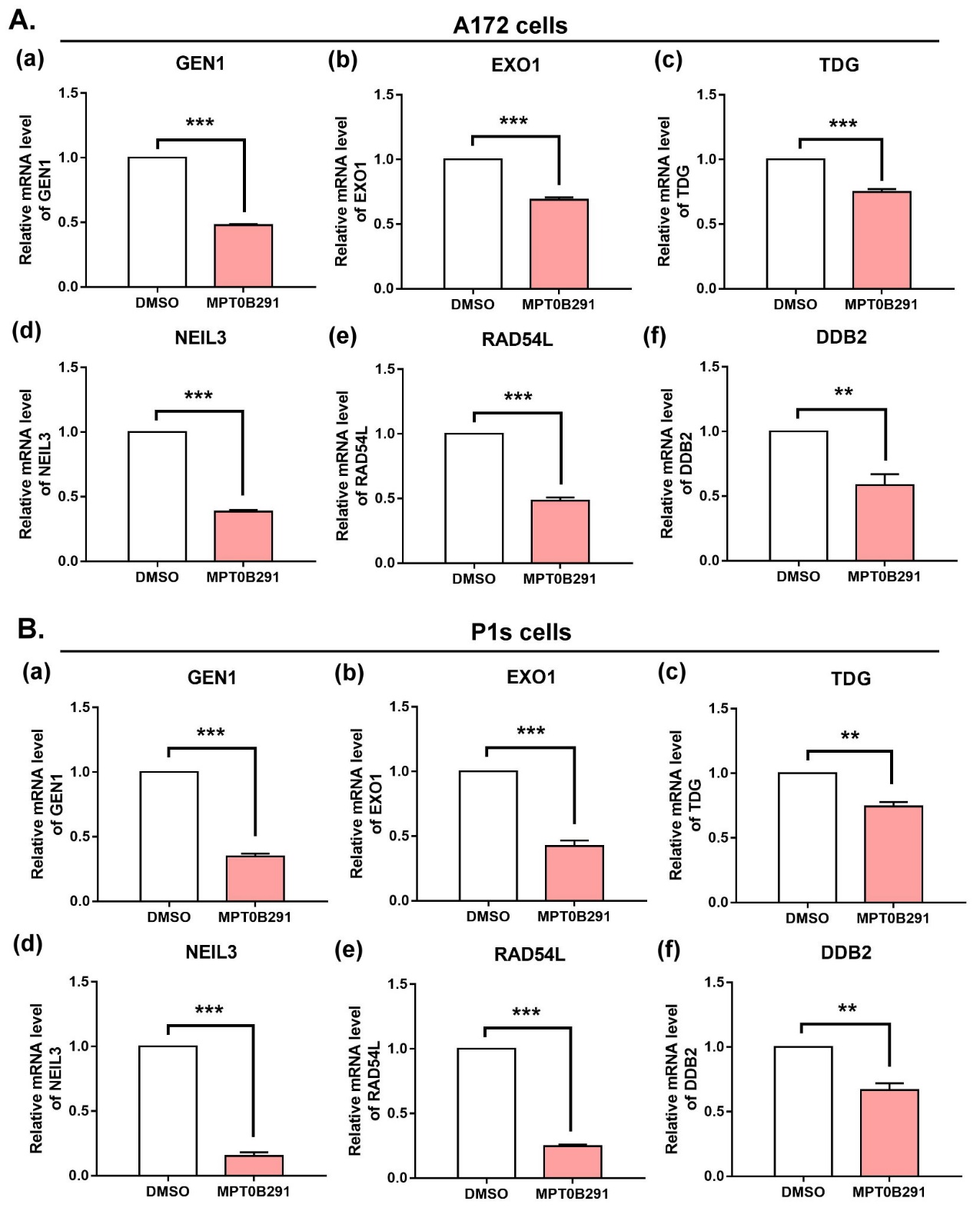


**Supplementary Figure S4. MPT0B291 decreases DDR genes expression in A172 and TMZ-resistant P1s cells.** (A) A172 or (B) P1s cells were treated with DMSO or 10 μM MPT0B291 for 24 h. The mRNA expression levels of GEN1 (panel a), EXO1 (panel b), TDG (panel c), NEIL3 (panel d), RAD54L (panel e), and DDB2 (panel f) in cells were analyzed by qPCR. Results from three independent experiments are shown.
