# Supplementary material for: Histone deacetylase 6 acts upstream of DNA damage response activation to support the survival of glioblastoma cells
Source: Cell Death Dis. 2021 Sep 28;12(10):884. doi: 10.1038/s41419-021-04182-w (PMC8479077; doi:10.1038/s41419-021-04182-w)
Supplement: Supplementary file 5 — Supplementary Figure S5 [file 41419_2021_4182_MOESM5_ESM.docx]

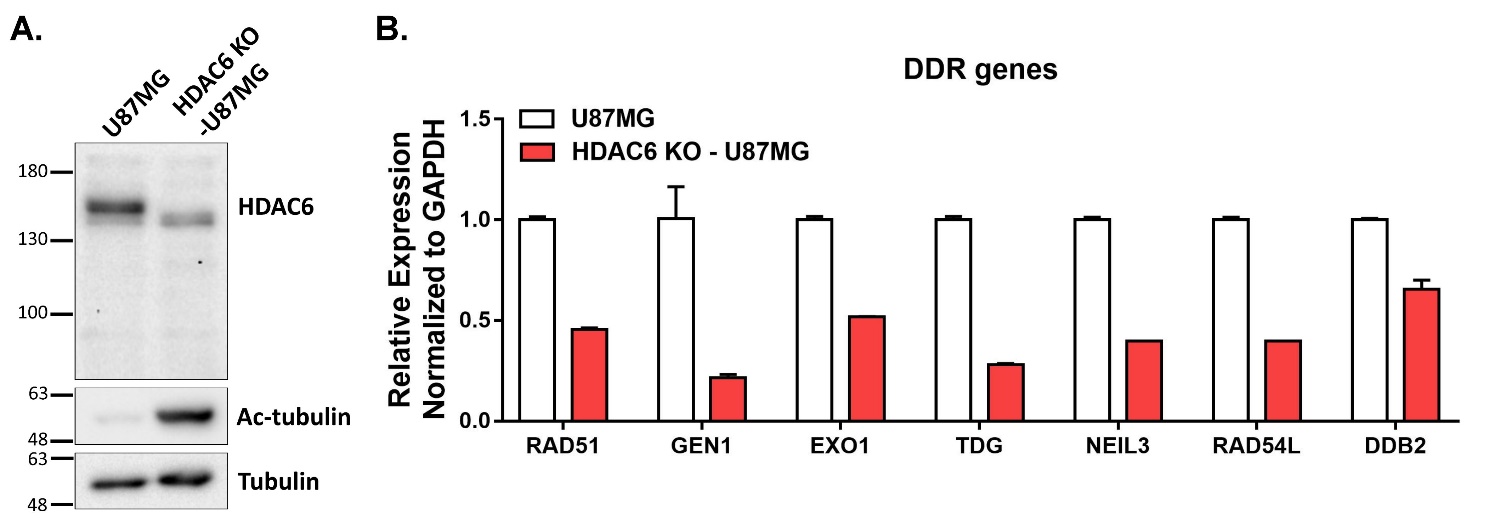


**Supplementary Figure S5. The expression of DDR genes was downregulated in HDAC6 KO - U87MG cells.** Cells of U87MG and HDAC6 KO - U87MG were harvested. (A) The protein levels of HDAC6, Ac-tubulin, and tubulin were analyzed by Western blotting. (B) The mRNA expression of RAD51, GEN1, EXO1, TDG, NEIL3, RAD54L, and DDB2 was analyzed by qPCR.
