# Supplementary material for: Histone deacetylase 6 acts upstream of DNA damage response activation to support the survival of glioblastoma cells
Source: Cell Death Dis. 2021 Sep 28;12(10):884. doi: 10.1038/s41419-021-04182-w (PMC8479077; doi:10.1038/s41419-021-04182-w)
Supplement: Supplementary file 6 — Supplementary Figure S6 [file 41419_2021_4182_MOESM6_ESM.docx]

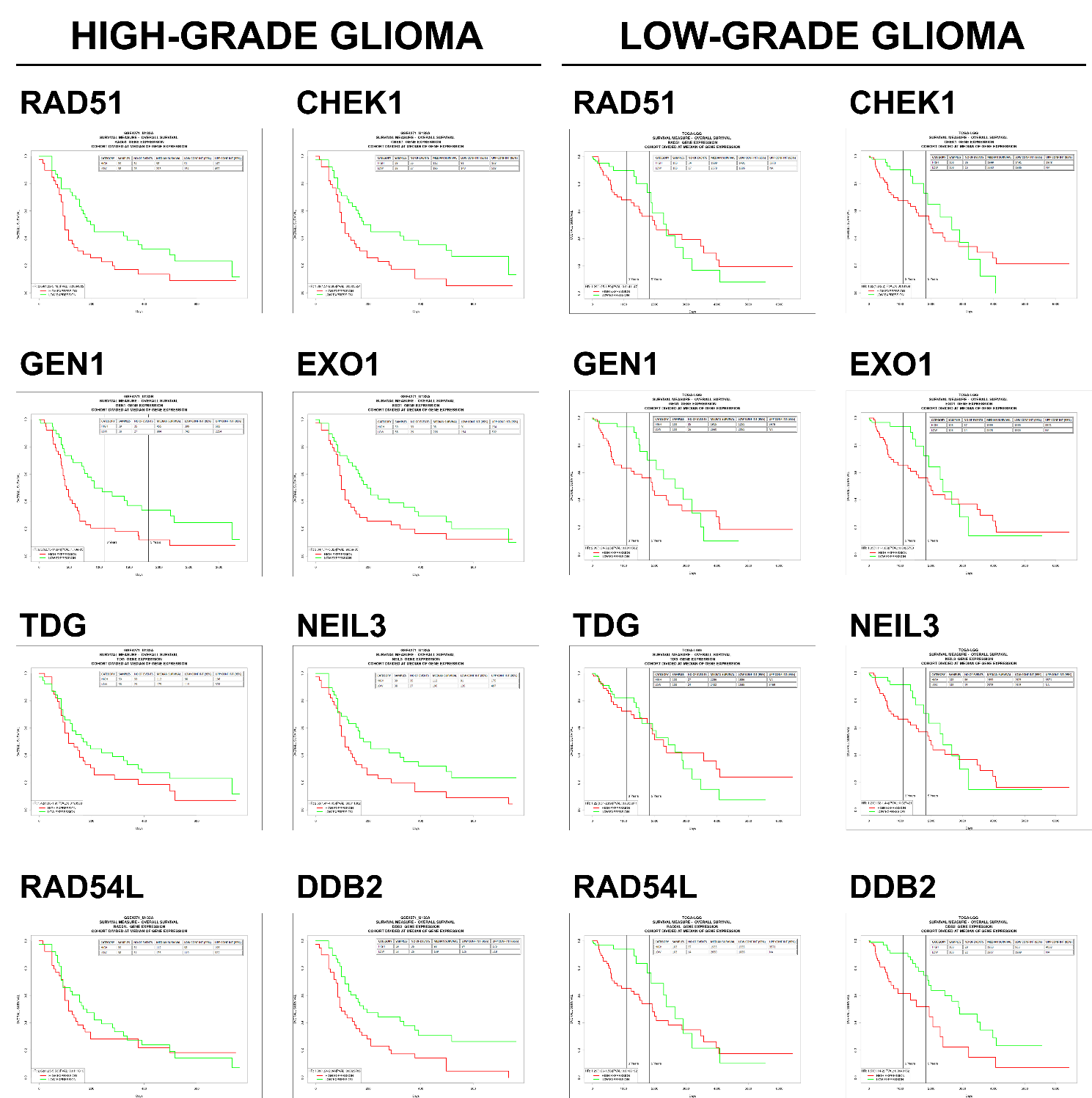


**Supplementary Figure S6. Kaplan–Meier curve analysis of indicated gene in patients with low-grade and high-grade glioma.** Data were obtained from PROGgeneV2 database.
