# Supplementary material for: Histone deacetylase 6 acts upstream of DNA damage response activation to support the survival of glioblastoma cells
Source: Cell Death Dis. 2021 Sep 28;12(10):884. doi: 10.1038/s41419-021-04182-w (PMC8479077; doi:10.1038/s41419-021-04182-w)
Supplement: Supplementary file 7 — Supplementary Figure S7 [file 41419_2021_4182_MOESM7_ESM.docx]

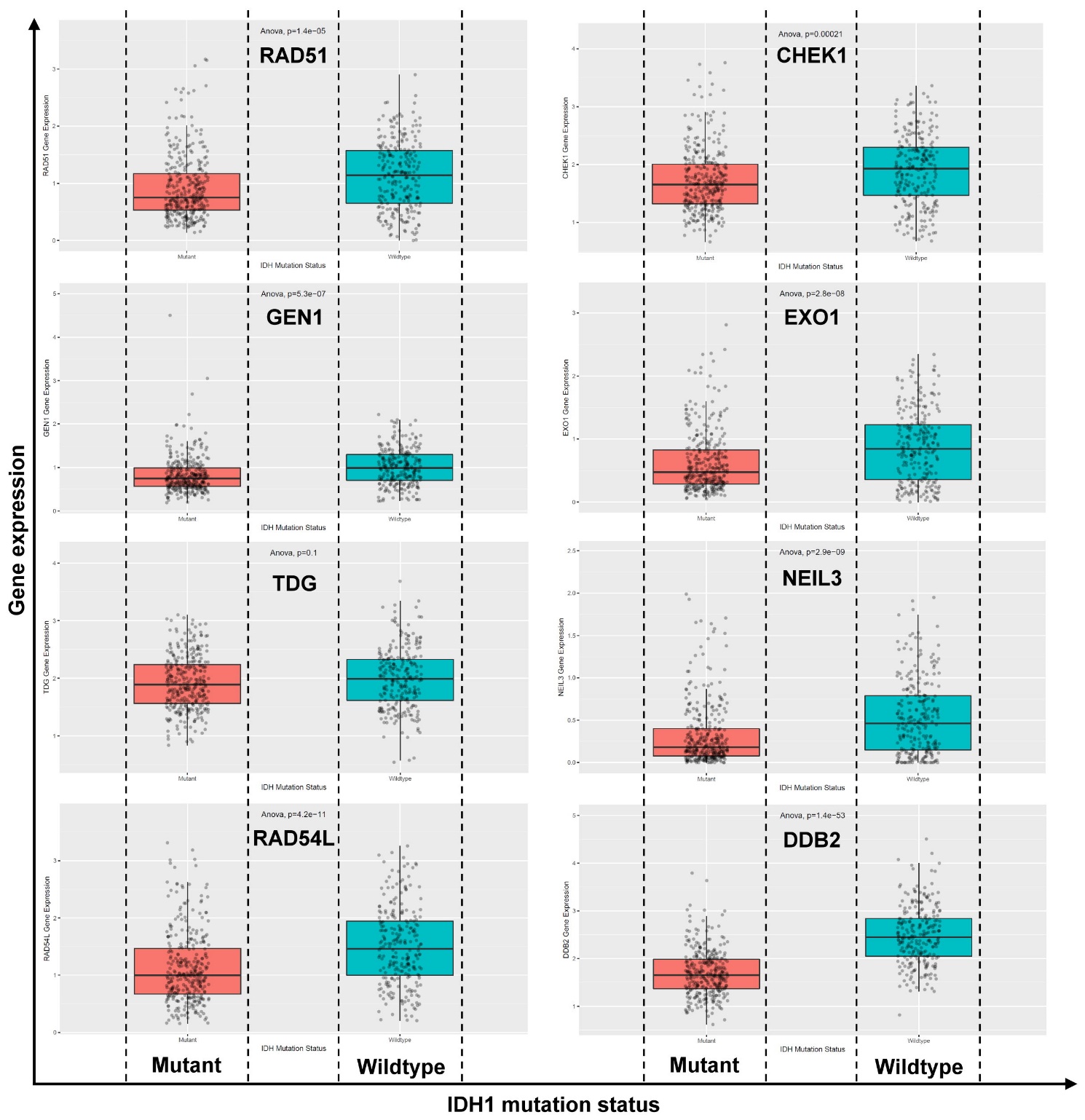


**Supplementary Figure S7. RAD51, GEN1, TDG, RAD54L, CHEK1, EXO1, NEIL3, and DDB2 presented low expression level in patients with IDH1 mutation.** Gene expression levels of indicated genes in patients with IDH1 wild-type or mutation status were analyzed using the CGGA database (<http://www.cgga.org.cn/>) with 693 glioma patients.
