# Supplementary material for: Histone deacetylase 6 acts upstream of DNA damage response activation to support the survival of glioblastoma cells
Source: Cell Death Dis. 2021 Sep 28;12(10):884. doi: 10.1038/s41419-021-04182-w (PMC8479077; doi:10.1038/s41419-021-04182-w)
Supplement: Supplementary file 8 — Supplementary Figure S8 [file 41419_2021_4182_MOESM8_ESM.docx]

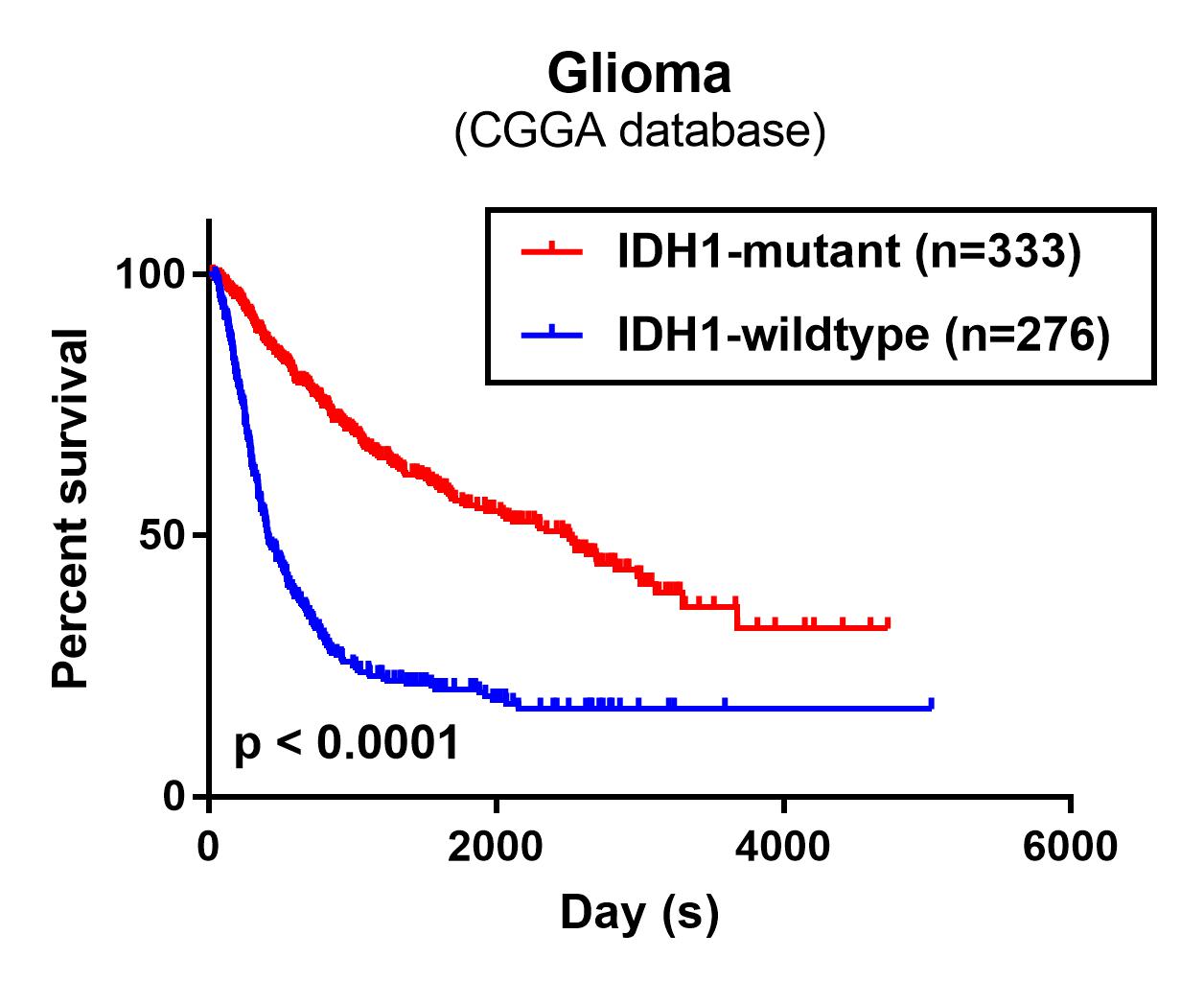


**Supplementary Figure S8. Glioma patients with IDH mutation exhibit better prognosis than IDH1 wild type.** Kaplan–Meier curve from mRNAseq_693 dataset of CGGA database. The IDH1 mutation status were grouped into IDH1-mutant or IDH1-wildtype. There are 51 patients without IDH1 information and 33 patients without overall survival information in mRNAseq_693 dataset of CGGA database.
