# Supplementary material for: Histone deacetylase 6 acts upstream of DNA damage response activation to support the survival of glioblastoma cells
Source: Cell Death Dis. 2021 Sep 28;12(10):884. doi: 10.1038/s41419-021-04182-w (PMC8479077; doi:10.1038/s41419-021-04182-w)
Supplement: Supplementary file 9 — Supplementary Table S1 [file 41419_2021_4182_MOESM9_ESM.docx]

**Supplementary Table S1. List of Sp1-regulated DDR genes.**

|  | ***Distance from TSS (bp)****^a^* | ***Log_2_ (ratio)*** | | | | |
| --- | --- | --- | --- | --- | --- | --- |
|  | *versus Input* | *versus Normal Brain* | | *MP-treated / DMSO* | | |
| ***Gene Symbol*** | ***Sp1-ChIP-seq in U87MG cells*** | ***TCGA-Primary GBM*** | ***TCGA-Recurrent GBM*** | | ***U87MG-R cells*** | ***P3-R cells*** |
| **RAD51** | **5.5** | **13.87** | **10.89** | | **-2.22** | **-1.05** |
| **CHEK1** | **227.5** | **5.02** | **4.78** | | **-2.03** | **-1.86** |
| **GEN1** | **51.5** | **2.24** | **2.44** | | **-1.43** | **-0.67** |
| **EXO1** | **34.5** | **12.3** | **9.77** | | **-0.73** | **0.85** |
| **TDG** | **210.5** | **1.89** | **1.94** | | **-0.72** | **-0.73** |
| **NEIL3** | **145** | **9.08** | **5.86** | | **-1.95** | **0.03** |
| **RAD54L** | **71.5** | **4.42** | **3.36** | | **-1.24** | **-0.55** |
| **DDB2** | **361** | **2.15** | **2.51** | | **-1.05** | **-0.28** |
| **FAAP24** | **130.5** | **3.58** | **5.39** | | **--^b^** | **-1.36** |
| **NUDT1** | **93.5** | **3.36** | **2.42** | | **-0.42** | **-0.2** |
| **RBBP8** | **425.5** | **5.91** | **6.2** | | **-0.24** | **-0.54** |
| **APEX2** | **186** | **5.04** | **3.94** | | **-0.21** | **-0.07** |
| **POLD1** | **131.5** | **2.83** | **2.48** | | **-0.05** | **-0.06** |
| **SLX1A** | **47** | **1.07** | **0.66** | | **--^b^** | **--^b^** |

^a^ The distance between TSS and Sp1-binding peaks from ChIP-seq analysis.

^b^ Not detected.

Red and green blocks represent upregulated and downregulated differentially expressed genes, respectively.
